# Supplementary material for: Targeted rRNA depletion enables efficient mRNA sequencing in diverse bacterial species and complex co-cultures
Source: mSystems. 2023 Oct 19;8(6):e00281-23. doi: 10.1128/msystems.00281-23 (PMC10734481; doi:10.1128/msystems.00281-23)
Supplement: Supplemental Information — Figures S1–S9; Tables S1–S3, S6, and S7; captions to Tables S4 and S5. [file msystems.00281-23-s0001.pdf]

Supplementary Information

Targeted rRNA depletion enables efficient mRNA sequencing in diverse bacterial species and complex co-cultures

Kellie A. Heom[a,b,*] , Chatarin Wangsanuwat[a,b,*,†], Lazarina V. Butkovich[a], Scott C. Tam[a], Annette R. Rowe[c], Michelle A. O'Malley[a,b,#] and Siddharth S. Dey[a,b,d,#]

[a] Department of Chemical Engineering, University of California Santa Barbara, Santa Barbara, CA

[b] Biological Engineering Program, University of California Santa Barbara, Santa Barbara, CA

[c] Biological Sciences, University of Cincinnati, Cincinnati, OH

[d] Neuroscience Research Institute, University of California Santa Barbara, Santa Barbara, CA

[†] Present address: Chatarin Wangsanuwat, Moderna, Inc., Cambridge, MA, U.S.A.

[#] Correspondence to: Michelle A. O'Malley (momalley@ucsb.edu) and Siddharth S. Dey (sdey@ucsb.edu).

Running title: Efficient mRNA-seq in non-model microbial systems

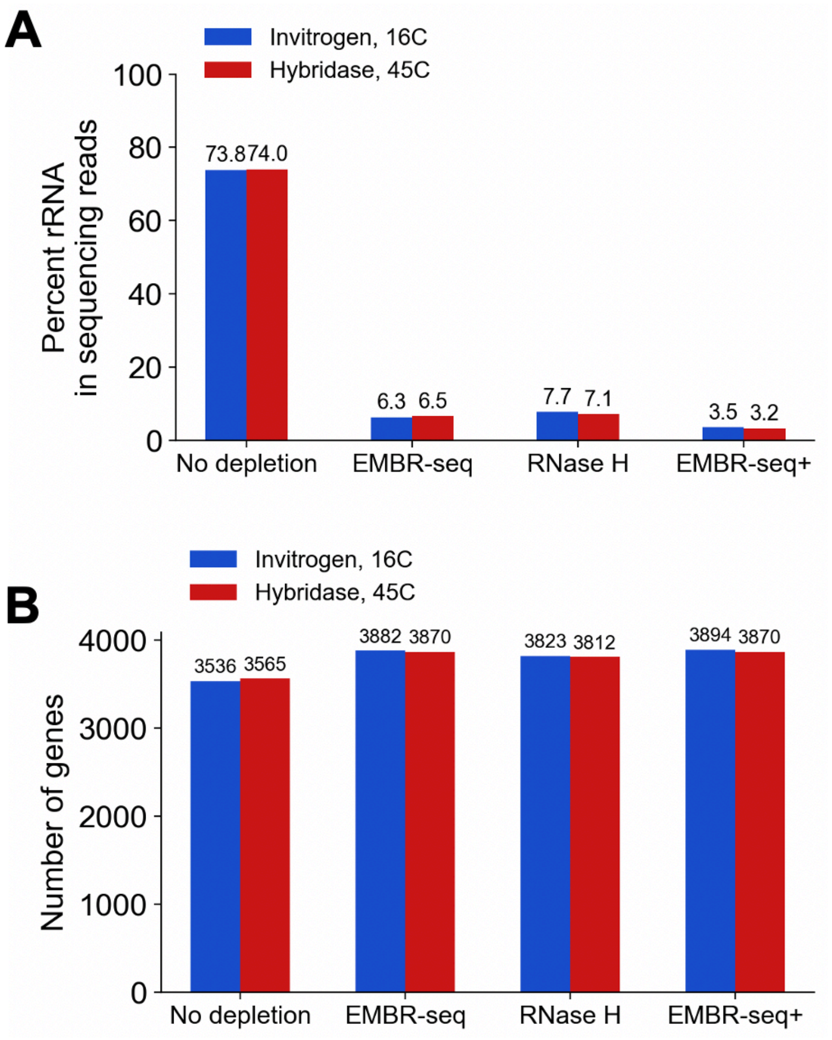

Supplementary Figure S1. Optimization of RNase H mediated degradation of rRNA. (**A**) Percent of sequencing reads mapping to rRNA in *E. coli* for four different library preparation methods using two different RNase H enzymes at different reaction temperatures (Invitrogen RNase H at 16°C and Hybridase Thermostable RNase H at 45°C). (**B**) The number of *E. coli* genes detected in each library. Both Invitrogen RNase H and Hybridase Thermostable RNase H show similar performance in these experiments.

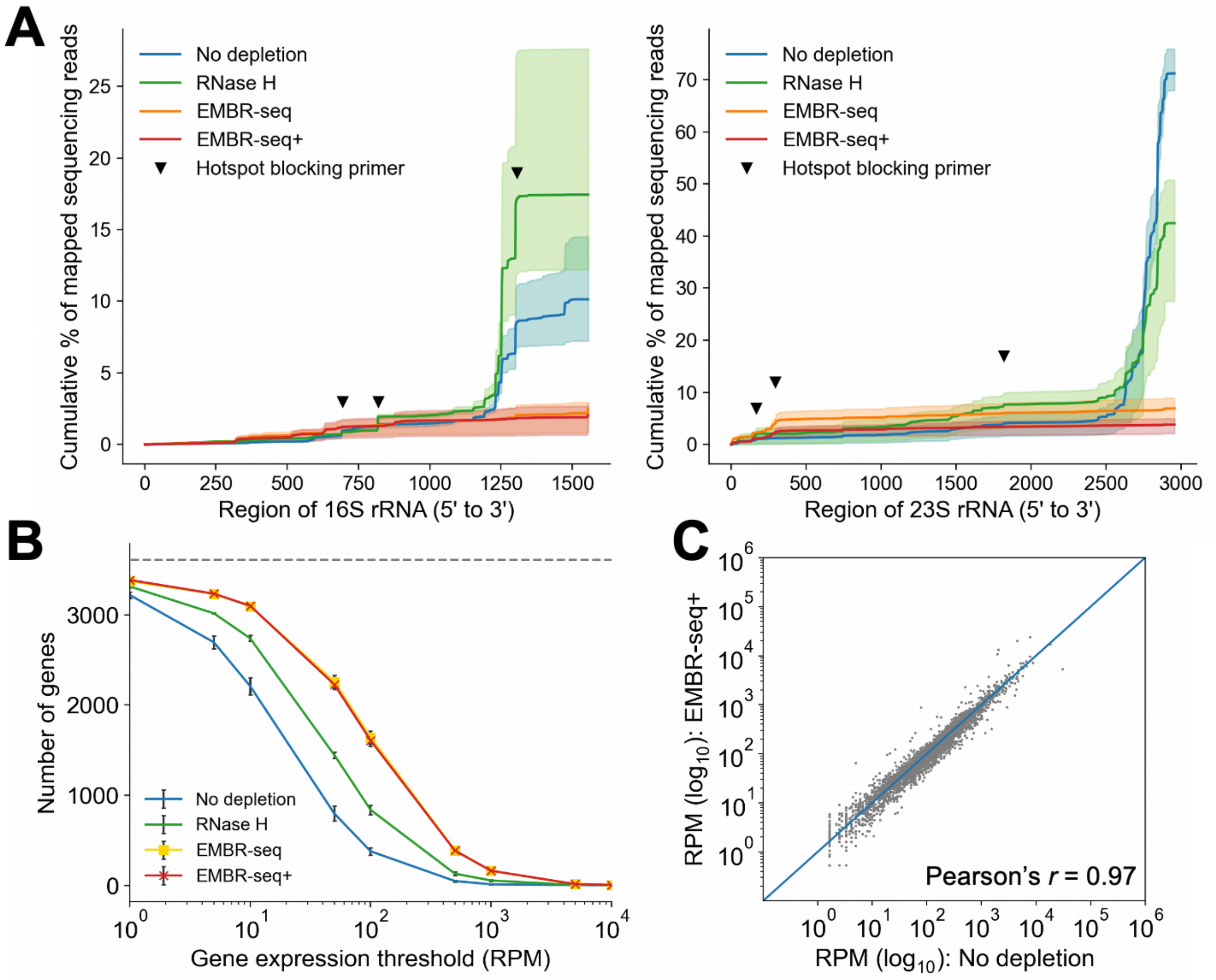

Supplementary Figure S2. Depletion of rRNA from *G. metallireducens* using EMBR-seq+. (**A**) Cumulative percentage of reads mapping along 16S and 23S rRNA of *G. metallireducens* for different depletion methods. Bold line indicates mean values and shaded regions indicate the minimum and maximum over three independent experiments. Inverted triangles indicate location of hotspots targeted by blocking primers. (**B**) Number of genes detected above different gene expression thresholds for *G. metallireducens*. Points indicate mean values and error bars indicate standard deviations over three independent experiments. (**C**) Correlation of gene expression between the “No depletion” and “EMBR-seq+” conditions for *G. metallireducens* (Pearson *r* = 0.97). RPM is computed

after removal of rRNA reads. The x- and y-coordinates of each point indicate mean values over three independent experiments in the two conditions. For consistent comparison across methods and to control for the variability in sequencing depth across samples, panels A and B show data that has been downsampled to 1 million sequencing reads.

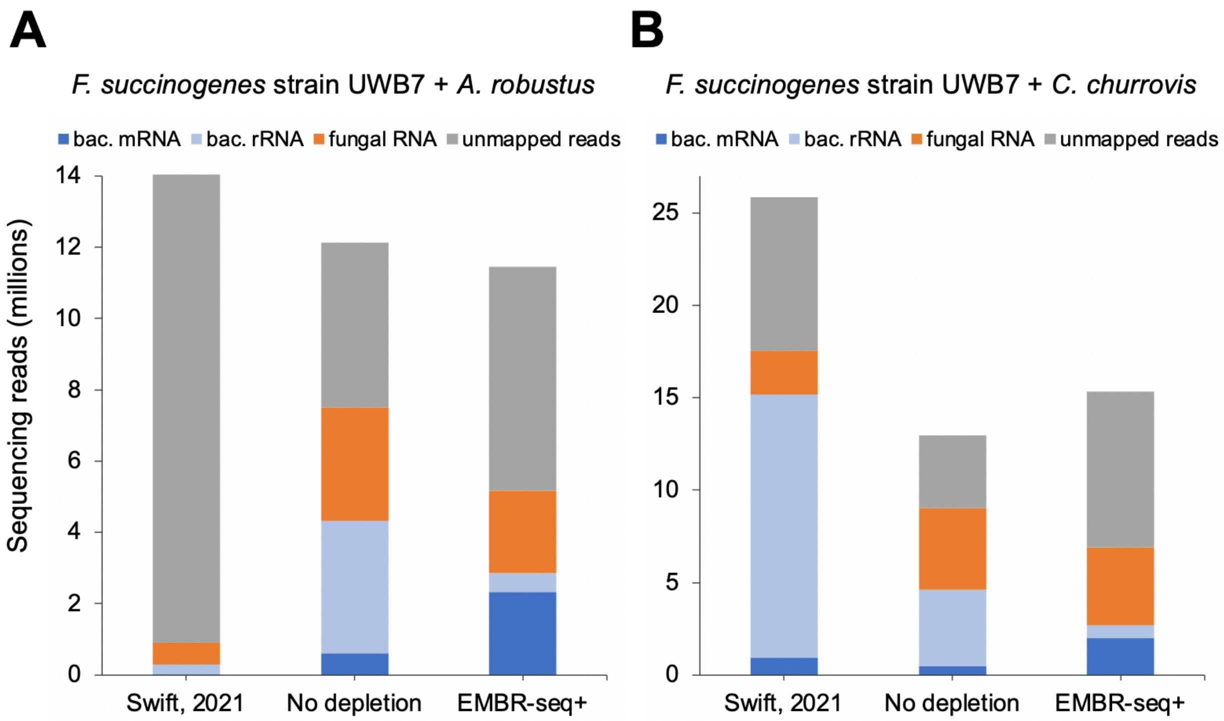

Supplementary Figure S3. Comparison of EMBR-seq+ sequencing data to a previous study using the same co-culture systems. “Swift, 2021” refers to data from a previous study that utilized the Ribo-Zero Gold rRNA Removal Kit (Epidemiology) for rRNA depletion. “No depletion” refers to libraries starting with “Poly-A depleted” RNA and prepared without EMBR-seq blocking primers or RNase H depletion. “EMBR-seq+” refers to “Poly-A depleted & RNase H treated” libraries. (**A**) RNA-seq libraries from co-cultures of *F. succinogenes* strain UWB7 with *A. robustus* grown on switchgrass. (**B**) RNA-seq libraries from co-cultures of *F. succinogenes* strain UWB7 with *C. churrovis* grown on switchgrass. All bars show mean values of sequencing reads over 3 or 4 replicates. To regenerate the data shown in “Swift, 2021”, raw FASTQ files were downloaded from the previous study (1) and mapped to the same reference transcriptomes used throughout this study (see *RNA sequencing and bioinformatics analysis* in *Materials and Methods*).

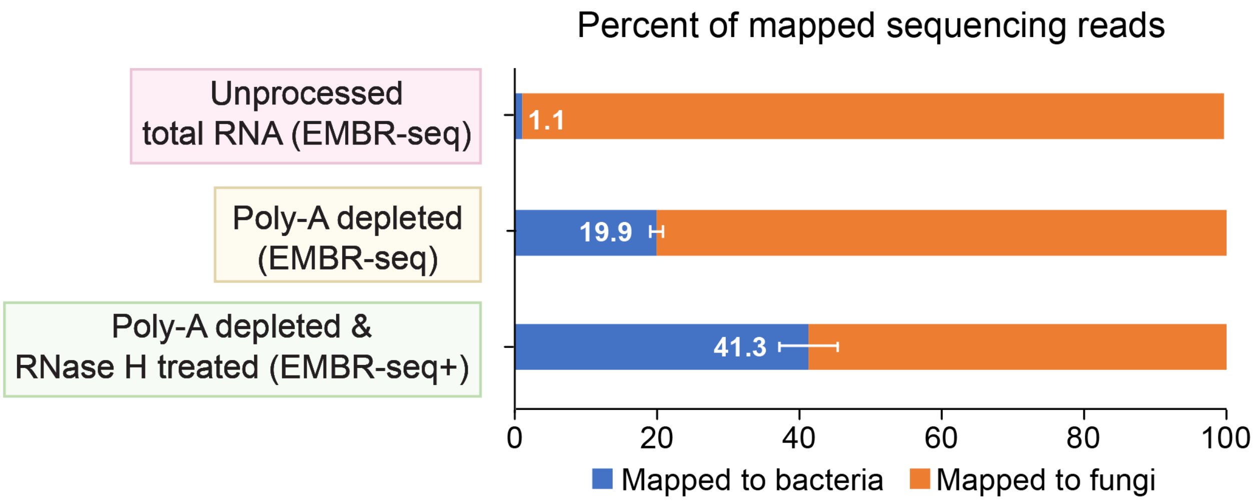

Supplementary Figure S4. Mapped RNA reads in libraries from co-cultures between *F. succinogenes* strain UWB7 and *C. churrovis*. Percentage of reads mapping to the bacterial and fungal genomes using the three strategies described in Figure 3A: total RNA isolated from co-culture pellets treated with EMBR-seq to remove bacterial rRNA ("Unprocessed total RNA (EMBR-seq)"), fungal poly-adenylated mRNA depleted total RNA is treated with EMBR-seq to remove bacterial rRNA ("Poly-A depleted (EMBR-seq)"), and "Poly-A depleted (EMBR-seq)" library additionally treated with RNase H to remove fungal and bacterial rRNA ("Poly-A depleted & RNase H treated (EMBR-seq+)"). For the "Unprocessed total RNA" library, *n* = 1. For "Poly-A depleted" and "Poly-A depleted & RNase H treated" libraries, *n* = 3. Bars indicate mean values and error bars indicate standard deviation from the mean.

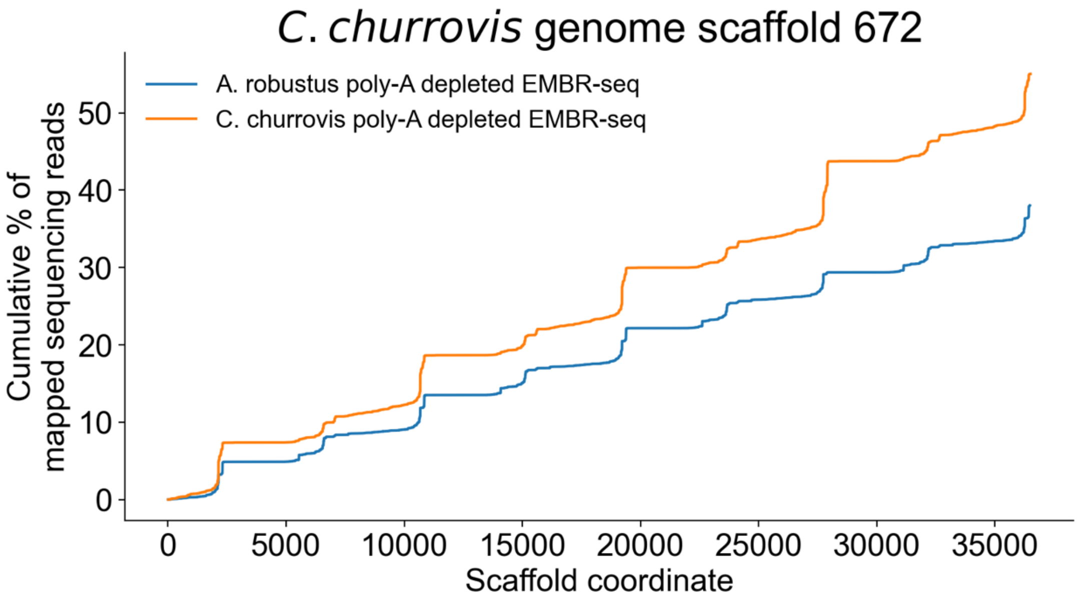

Supplementary Figure S5. Mapping locations of sequencing reads from "Poly-A depleted" EMBR-seq libraries along *C. churrovis* scaffold 672. Cumulative percentage of reads mapping to *C. churrovis* scaffold 672 from co-culturing *F. succinogenes* strain UWB7 with *C. churrovis* or *A. robustus* (n = 1). By permitting non-unique mapping, a repetitive pattern was observed along the scaffold. EMBR-seq data from the co-culture of *F. succinogenes* strain UWB7 with *A. robustus* also exhibited high mappability on the *C. churrovis* genome, implying high sequence conservation between the two fungal strains on this scaffold.

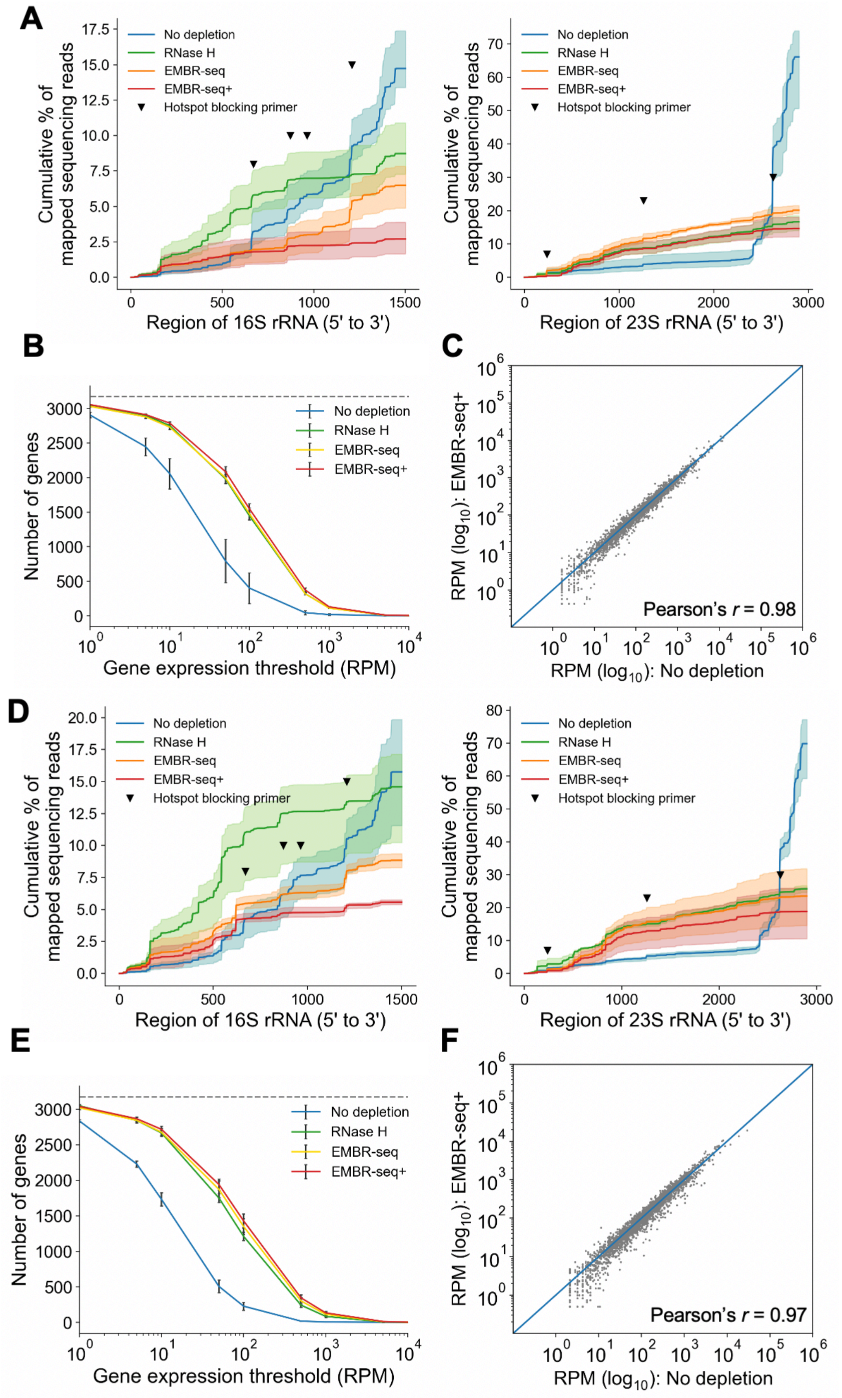

Supplementary Figure S6. EMBR-seq+ efficiently depletes bacterial rRNA from co-cultures of *F. succinogenes* strain UWB7 with *A. robustus* or *C. churrovis*. (**A,D**) Cumulative percentage of reads mapping to 16S and 23S rRNA of *F. succinogenes* strain UWB7 cultured with *A. robustus* (panel A) or *C. churrovis* (panel D) for different depletion

methods. Bold lines indicate mean values and shaded regions indicate the minimum and maximum of three independent experiments. Inverted triangles indicate location of hotspots targeted by blocking primers. (**B,E**) Number of genes detected above different gene expression thresholds for *F. succinogenes* strain UWB7 cultured with *A. robustus* (panel B) or *C. churrovis* (panel E). Points indicate mean values and error bars indicate standard deviations over three independent experiments. (**C**,**F**) Correlation of gene expression between the “No depletion” and “EMBR-seq+” conditions for *F. succinogenes* strain UWB7 (Pearson’s $r = 0.98$ for co-culture with *A. robustus* (panel C) and $r = 0.97$ for co-culture with *C. churrovis* (panel F)). RPM is computed after removal of rRNA reads. The x- and y-coordinates of each point indicate mean values over three independent experiments in the two conditions. For consistent comparison across methods and to control for the variability in sequencing depth across samples, panels A, B, D, and E show data that has been downsampled to 1 million sequencing reads.

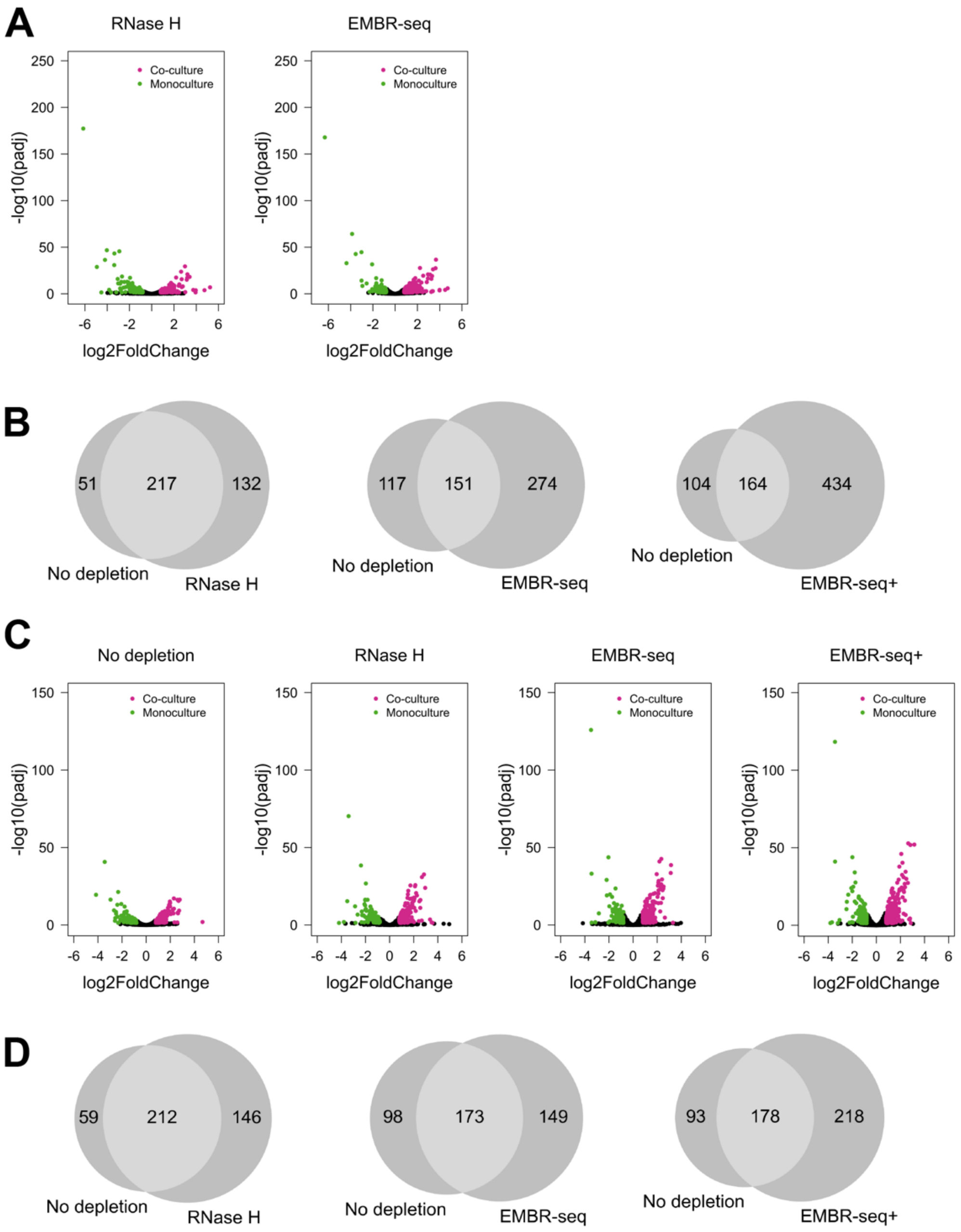

Supplementary Figure S7. Summary of differentially expressed genes for *F. succinogenes* strain UWB7 grown in monoculture *vs.* co-culture across different depletion methods. (**A,C**) Volcano plots of differentially expressed genes for *F. succinogenes* strain UWB7 grown in monoculture *vs.* co-culture (with *A. robustus* (panel A) or *C. churrovis* (panel C)), for the "No depletion" condition and other rRNA depletion

methods. In these plots, *n* = 3 for both monoculture and co-culture conditions and all depletion methods. Colored data points indicate differentially expressed genes, determined by thresholds of $|\log_2(Fold\ Change)| > 0.8$, $P_{adj} < 0.05$, and RPM > 2. Green and pink data points correspond to genes upregulated in monoculture and co-culture, respectively. (**B**,**D**) Venn diagrams show the number of differentially expressed genes identified between monoculture and co-culture (with *A. robustus* (panel B) or *C. churrovis* (panel D))_conditions that overlap between the "No depletion" condition and different rRNA depletion methods.

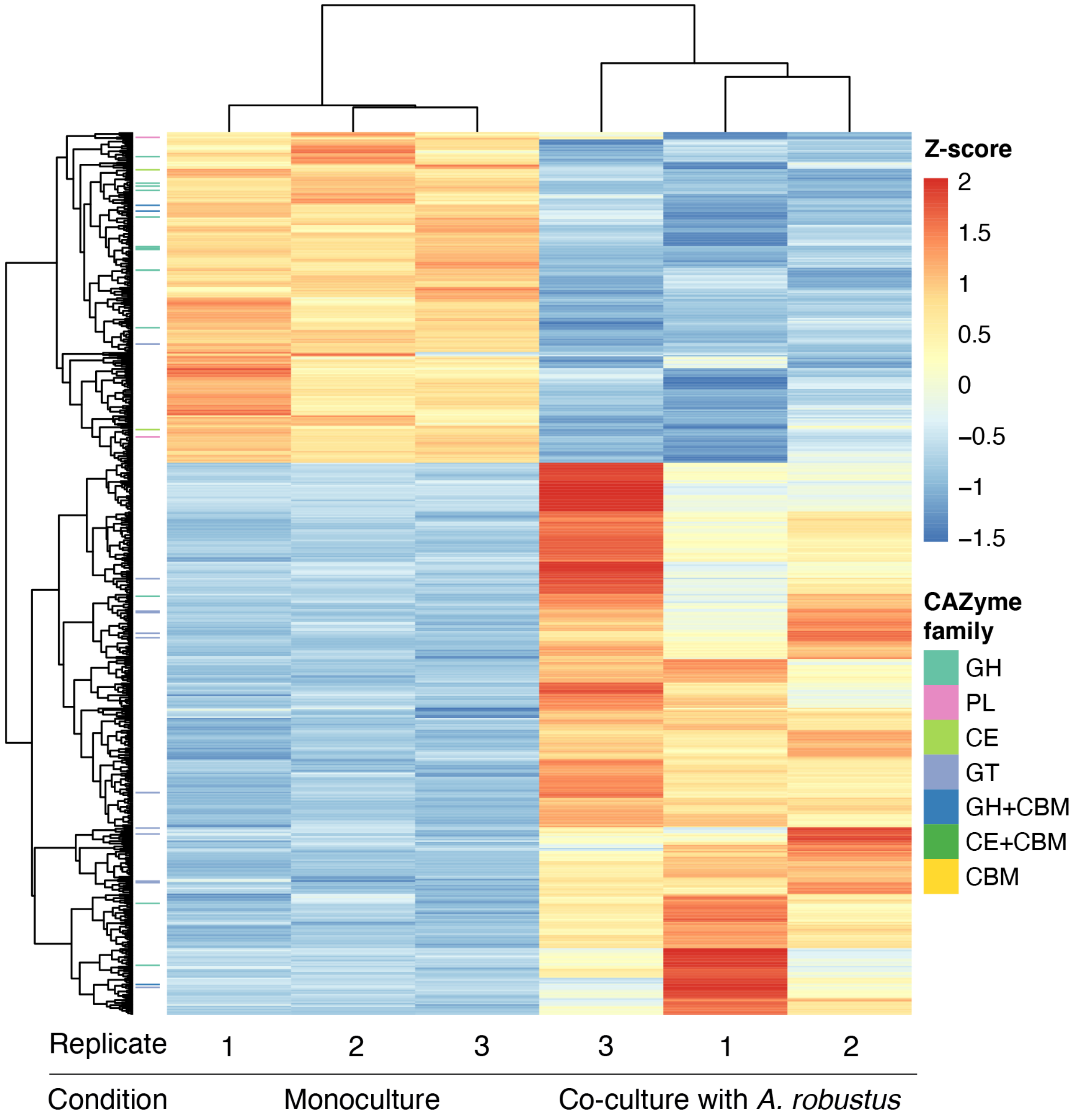

Supplementary Figure S8. Heatmap of all differentially expressed genes for *F. succinogenes* strain UWB7 grown in monoculture *vs.* in co-culture with *A. robustus*. Heatmap shows all the differentially expressed bacterial genes for three independent replicates when *F. succinogenes* strain UWB7 is grown in monoculture *vs.*

in co-culture with *A. robustus*. Genes are colored on the left side of the heatmap based on the CAZyme family they are associated with.

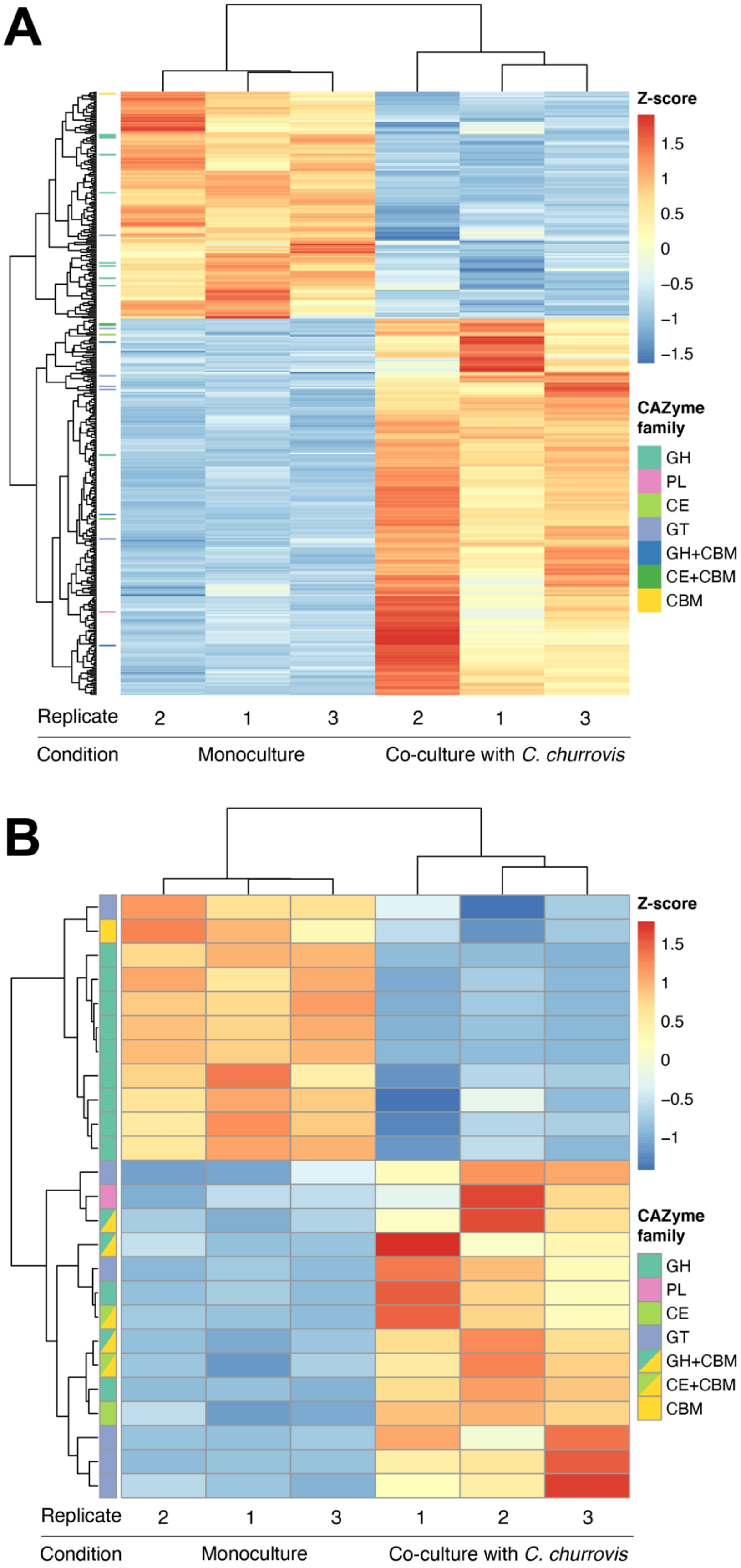
A
Z-score
1.5
1
0.5
0
−0.5
−1
−1.5
CAZyme family
GH
PL
CE
GT
GH+CBM
CE+CBM
CBM
Replicate 2 1 3 2 1 3
Condition Monoculture Co-culture with C. churrovis
B
Z-score
1.5
1
0.5
0
−0.5
−1
CAZyme family
GH
PL
CE
GT
GH+CBM
CE+CBM
CBM
Replicate 2 1 3 1 2 3
Condition Monoculture Co-culture with C. churrovis

Supplementary Figure S9. Heatmaps of differentially expressed genes when *F. succinogenes* strain UWB7 is grown in monoculture *vs.* in co-culture with *C. churrovis*. (**A**) Heatmap showing all differentially regulated genes for experiments performed in triplicate. (**B**) Heatmap showing only differentially regulated CAZymes for experiments performed in triplicate. Genes are colored on the left side of the heatmap based on the CAZyme family they are associated with.

Supplementary Table S1. Comparison between various rRNA depletion approaches.

Supplier or publication	Working principle	Cost/sample (approx.)	Number of primers per species	Percent rRNA left	Notes
This work (EMBR-seq+)	Blocked reverse transcription & RNase H digestion	$8.29	17-20	1-10% (monocultures), 20% (co-cultures)	
EMBR-seq (2)	Blocked reverse transcription	$0.39 (with hotspot BPs)	3-10	3-20% (monocultures)	Tested with and without hotspot primers
Kraus *et al*, 2019 (3)	Oligo-based rRNA pull-down	N/A	12	<5%	
Culviner *et al*, 2020 (4)	Oligo-based rRNA pull-down	$10	21	~20-25%	17 of the probes were optimized to target 8 species simultaneously
Huang *et al*, 2020 (5)	RNase H digestion	$12.94	90	<5% to ~25%	
Engelhardt *et al*, 2020 (6)	RNase H digestion	N/A	90	<5%	Targets rRNA as well as transfer-messenger RNA (tmRNA)
Choe *et al*, 2021 (7)	RNase H digestion	$10	60-130+	<10%	
Prezza *et al*, 2020 (8)	Cas9-mediated cleavage of rRNA-derived cDNA	$3-7	120	~10-50%	
Ribo-Zero Plus Microbiome rRNA Depletion Kit (Illumina Cat # 20072062)	RNase H digestion	N/A; similar RiboZero product is $47	/	~20-30%	Targets common gut bacteria, including those in ATCC MSA-2002, MSA-2005, and MSA-2006.
RiboMinus™ Bacteria 2.0 Transcriptome Isolation Kit (Thermo Fisher Cat # A47335)	Oligo bead-based pull down	$82	>150	<10%	Targets 76 bacterial species
rRNA Depletion Kit for Bacteria (NEBNex Cat # E7850X)	RNase H digestion	$33	/	~1-5% for several species, but varies	Compatible with ~60 bacterial species with varying efficiency (NEB & User-reported data)

Supplementary Table S2. List of all primers used in EMBR-seq+, including primers for library preparation associated with CEL-seq2, EMBR-seq blocking primers, and RNase H probes. Numbers in the names of the hotspot primers and probes indicate the transcript coordinate of the rRNA hotspot.

Primer name	Primer sequence (from 5' to 3')
Primers associated with CEL-seq2	
CEL-seq2 reverse transcription	GCCGGTAATACGACTCACTATAGGGAGTTCTACAGTCCGACGATCNNNNNNBBBBBBTTTTTTTTTTTTTTTTTTTTTTTTV<br>where 'BBBBBB' is the sample barcode. The barcodes used in this study were:<br>AGACTC<br>AGCTTC<br>CATGAG<br>CAGATC<br>TCACAG
Random hexamer RT primer for library preparation	GCCTTGGCACCCGAGAATTCCANNNNNN
Illumina RPI PCR primer (see TruSeq Small RNA kit)	AATGATACGGCGACCACCGAGATCTACACGTTCAGAGTTCTACAGTCCGA
Illumina RPI[1-48] PCR primers (see TruSeq Small RNA kit for RPI sequences)	CAAGCAGAAGACGGCATACGAGAT[6-bpRPI]GTGACTGGAGTTCCTTGGCACCCGAGAATTCCA
E. coli EMBR-seq blocking primers	
16S 3'-end	TAAGGAGGTGATCCAACCGCAGGTTCCCCT
23S 3'end	AAGGTTAAGCCTCACGGTTCATTAGTACCG
5S 3'end	ATGCCTGGCAGTTCCCTACTCTCGCATGGG
16S 107	GGCACATCCGATGGCAAGAGGCCCGAAGGT
16S 682	TCCTGTTTGCTCCCCACGCTTTCGCACCTG
16S 1241	CCGTGGCATTCTGATCCACGATTACTAGCGATTCCG
23S 375	CGCCTTTCCCTCACGGTACTGGTTCACTATCGG

23S 1421	TTGCTTCAGCACCGTAGTGCCTCGTCATCA
23S 1641	GCAGCCAGCTGGTATCTTCGACTGATTTCAGC
E. coli RNase H probes	
16S 3'end	AGTCGTAACAAGGTAACCGTAGGGGAACCTGCGGTTGGATCACCTCCTTA
23S 3'end	CAGCGATGCGTTGAGCTAACCGGTACTAATGAACCGTGAGGCTTAACCTT
16S 107	TCGCAAGACCAAAGAGGGGGACCTTCGGGCCTCTTGCCATCGGATGTGCC
16S 682	CAGGTGCGAAAGCGTGGGGAGCAAACAGGATTAGATACCCTGGTAGTCCA
16S 1241	CGACTCCATGAAGTCGGAATCGCTAGTAATCGTGGATCAGAATGCCACGG
23S 375	CCGATAGTGAACCAGTACCGTGAGGGAAAGGCGAAAAGAACCCCGGCGAG
23S 1421	GGAAAATCAAGGCTGAGGCGTGATGACGAGGCACTACGGTGCTGAAGCAA
23S 1641	AAGCGACTTGCTCGTGGAGCTGAAATCAGTCGAAGATACCAGCTGGCTGC
G. metallireducens EMBR-seq blocking primers	
16S 3'end	AAAGGAGGTGATCCAGCCGCAGGTTCCCCT
23S 3'end	ATGATCAAGCCTCACGGCCGATTAGTACCGG
5S 3'end	TCCCGGCGGCGACCTACTTTCCCACACAGC
16S 694	GGCGTGGACTACCAGGGTATCTAATCCTGTTTGCTC
16S 819	TTGTGCGGGCCCCCGTCAATTCCTTTGAGT
16S 1306	GTCGACTCCCGTGGTGTGACGGGCGGTGTG
23S 169	CCCGGCAACACCGTAGTATCACCGGTTTGG
23S 296	GTCTCGCGGTACTCGGGGACACCCTAGGGT
23S 1818	GGGCTTCGGTTCAAAGCTTCGCTTGCGCTA
G. metallireducens RNase H probes	
16S 3'end	AGTCGTAACAAGGTAGCCGTAGGGGAACCTGCGGCTGGATCACCTCCTTT
23S 3'end	CAGCAATGCGCTCAGCTGACCGGTACTAATCGGCCGTGAGGCTTGATCAT
5S 3'end	CCGATGATACTGCACGGGTAGCTGTGTGGGAAAGTAGGTCGCCGCCGGGA
16S 694	CGCGAAAGCGTGGGGAGCAAACAGGATTAGATACCCTGGTAGTCCACGCC

16S 819	AGTACGGCCGCAAGGCTAAAACTCAAAGGAATTGACGGGGGCCCGCACAA
16S 1306	ATACGTTCCCGGGCCTTGTACACACCGCCCGTCACACCACGGGAGTCGAC
23S 169	GCGAGCGAAAGCGGAACAGCCCAAACCGGTGATACTACGGTGTTGCCGGG
23S 296	CGTATGCGAAACCTTGATTCACCCTAGGGTGTCCCCGAGTACCGCGAGAC
23S 1818	GGAAGGTTAAGGGGATTTGTTAGCGCAAGCGAAGCTTTGAACCGAAGCCC
F. succinogenes strain UWB7 EMBR-seq blocking primers	
16S 3’end	TAAAGGAGGTAATCCAGCCGCACCTTCCGG
23S 3’end	AAAAGCGTCTCACGGGCTATTAGTACCGCTCG
5S 3’end	ATCCAGCGGCGACCTACTCTCCCGGGCCCG
16S 670	GCATTGTTTACGGTGCGGACTACCAGGGTATCT
16S 871	AACGGCGATCTCTCGCCGCGGCGTTCCCAT
16S 964	GGGTTGCGCTCGTTGCGGGACTTAACCCAA
23S 239	TCTTCGACGGACATCAGATTCCAGATCGCTACAGG
23S 1258	CGCCTGGTCGTCATCGGCTACGCCTCTCGG
23S 2627	ATGCCTTCAGCGTTTCTCCAATCCGGACATG
F. succinogenes strain UWB7 RNase H probes	
16S 3’end	GTCGTAACAAGGTAGCCGTACCGGAAGGTGCGGCTGGATTACCTCCTTTA
23S 3’end	CGTGGCGACACGTTGAGCCGAGCGGTACTAATAGCCCGTGAGACGCTTTT
5S 3’end	CGCCGATGGTACCTGGCCCCCGGGCCCGGGAGAGTAGGTCGCCGCTGGAT
16S 670	GGGTAGCAAACAGGATTAGATACCCTGGTAGTCCGCACCGTAAACAATGC
16S 871	GAACCTTACCAGGGTTTGACATGGGAACGCCGCGGCGAGAGATCGCCGTT
16S 964	TCAGCTCGTGTCGTGAGATGTTGGGTTAAGTCCCGCAACGAGCGCAACCC
16S 1209	TCTGCAACTCGACTCCGTGAAGCTGGAATCGCTAGTAATCGTGGGTCAGC
23S 239	ACAGCAGGTGACAGGCCTGTAGCGATCTGGAATCTGATGTCCGTCGAAGA
23S 1258	AGTCGGGATCTAAGACGAGGCCGAGAGGCGTAGCCGATGACGACCAGGCG
23S 2627	AGCGGCACTGCCGGGTAGCCATGTCCGGATTGGAGAAACGCTGAAGGCAT

C. churrovis RNase H probes	
28S 3’end #1 (2196)	TTGTTCCAATTCCGGAATCATACAAGAGGACTCCGAGTTAGGCGCGGAAG
28S 3’end #2 (2307)	GCAGACGACTTAAATTTAGAACGAGGTATTGTAAGCAGTAGAGTAGCCTTGTT
18S 3’end #1 (6581)	TGCCCTTTGTACACACCGCCCGTCGCTACTACCGATTGAATGGCTTAGTG
18S 3’end #2 (6706)	GGAAGTAAAAGTCGTAACAAGGTTTCCGTAGGTGAACCTGCGGAAGGATC
5.8S 3’end (7057)	GTGTGAATTGCAGAATACGTGAATCATCGAATCTTCGAACGCATATTGCA

Supplementary Table S3. Pearson's *r* correlation values between RPM counts for RNA-seq libraries prepared with different methods (average over 3 replicates) and between individual replicates.

E. coli	
**Pairing**	**Pearson's *r* (log10 of counts)**
Avg N vs Avg R	0.98265225
Avg N vs Avg E	0.95167825
Avg N vs Avg E+	0.95234564
Avg E vs Avg E+	0.99065287
N1 vs N2	0.9516501
N1 vs N3	0.94731079
N2 vs N3	0.95406484
R1 vs R2	0.97926869
R1 vs R3	0.97496608
R2 vs R3	0.97721349
E1 vs E2	0.98201767
E1 vs E3	0.97846141
E2 vs E3	0.97914359
E+1 vs E+2	0.98568803
E+1 vs E+3	0.98279637
E+2 vs E+3	0.98175982

G. metallireducens	
**Pairing**	**Pearson's *r* (log10 of counts)**
Avg N vs Avg R	0.99024853
Avg N vs Avg E	0.97250812
Avg N vs Avg E+	0.97435751
Avg E vs Avg E+	0.98958999
N1 vs N2	0.94028986
N1 vs N3	0.94056497
N2 vs N3	0.98147716
R1 vs R2	0.95841967
R1 vs R3	0.96243051
R2 vs R3	0.9815216
E1 vs E2	0.9199076
E1 vs E3	0.91220263
E2 vs E3	0.96130948
E+1 vs E+2	0.93009685
E+1 vs E+3	0.91913965
E+2 vs E+3	0.96298596

***F. succinogenes* strain UWB7, monoculture**	
**Pairing**	**Pearson's *r* (log10 of counts)**
Avg N vs Avg R	0.98477931
Avg N vs Avg E	0.94025124
Avg N vs Avg E+	0.93949563
Avg E vs Avg E+	0.99348052
N1 vs N2	0.95929008
N1 vs N3	0.94538248
N2 vs N3	0.9636034
R1 vs R2	0.9788226
R1 vs R3	0.96883094
R2 vs R3	0.97944562
E1 vs E2	0.98158273
E1 vs E3	0.98713194
E2 vs E3	0.98529399
E+1 vs E+2	0.98704526
E+1 vs E+3	0.98725156
E+2 vs E+3	0.98600873

**F. succinogenes strain UWB7 + A. robustus, co-culture**	
**Pairing**	**Pearson's *r* (log10 of counts)**
Avg N vs Avg R	0.98751771
Avg N vs Avg E	0.98711161
Avg N vs Avg E+	0.98242714
Avg E vs Avg E+	0.98573729
N1 vs N2	0.93065604
N1 vs N3	0.91587555
N2 vs N3	0.95371916
R1 vs R2	0.95860025
R1 vs R3	0.94876217
R2 vs R3	0.97122295
E1 vs E2	0.95340603
E1 vs E3	0.93497145
E2 vs E3	0.96365285
E+1 vs E+2	0.96833389
E+1 vs E+3	0.9564747
E+2 vs E+3	0.97353485

**F. succinogenes strain UWB7 + C. churrovis, co-culture**	
**Pairing**	**Pearson's *r* (log10 of counts)**
Avg N vs Avg R	0.98496094
Avg N vs Avg E	0.97366127
Avg N vs Avg E+	0.97429749
Avg E vs Avg E+	0.989128
N1 vs N2	0.92430954
N1 vs N3	0.94514701
N2 vs N3	0.96064716
R1 vs R2	0.95900951
R1 vs R3	0.97586658
R2 vs R3	0.98192151
E1 vs E2	0.95873219
E1 vs E3	0.96924318
E2 vs E3	0.9782951
E+1 vs E+2	0.97139885
E+1 vs E+3	0.98166885
E+2 vs E+3	0.9837209

Supplementary Table S4. Results of differential gene expression analysis for comparisons between *F. succinogenes* strain UWB7 grown in monoculture *vs.* co-culture, using “No depletion”, “EMBR-seq”, “RNase H”, and “EMBR-seq+” libraries. For EMBR-seq+ results, predicted CAZyme annotations from dbCAN2 are also included (columns U-Z where applicable). In the table, columns I-N (named “M1”…“C3”) show RPM for each gene (calculated as raw counts * 10^6 / sum of raw counts). Columns P and Q (“UR_Mono” and “UR_Coc”) indicate the upregulated genes before filtering in monoculture and co-culture conditions, respectively. Column R (named “Alpha”) indicates genes that are differentially expressed between the “No Depletion” and “EMBR-seq+” libraries, and are filtered out in column S (named “Beta”). A total of 239 genes are filtered out. In column S, a value of 0 indicates a gene that is not differentially expressed or is differentially expressed but filtered out, -1 indicates a gene that is upregulated in co-culture, and 1 indicates a gene that is upregulated in monoculture. This supplementary table is provided as a separate Excel file.

Supplementary Table S5. Select bacterial stress response genes upregulated in co-culture with anerobic fungi *A. robustus* or *C. churrovis*. Individual groups of genes of interest were identified by looking at COG annotations, KEGG orthology annotations, and the CAZyme gene cluster finder (CGC). In the table, columns H-M (named “M1”…“C3”) show RPM for each gene (calculated as raw counts * 10^6 / sum of raw counts). This supplementary table is provided as a separate Excel file.

Supplementary Table S6. Comparison of bacterial transporters upregulated in co-culture in this work and those reported in Table 2 of Swift *et al.* (1). Columns A-C contain *F. succinogenes* strain UWB7 genes reported in Table 2 of Swift *et al*. as upregulated in co-culture with *C. churrovis*. Columns D-L contain $\log_2$(Fold Change) and Padj values from our datasets (*F. succinogenes* strain UWB7 co-culture with *C. churrovis* (columns D-G) and *A. robustus* (I-L)) for the genes of interest. For our datasets, a threshold of $P_{adj} < 0.1$ and $|\log_2(Fold\ Change)| > 0.5$ was used to determine if a gene was differentially expressed. Note: In EMBR-seq+ results, negative log2 fold change indicates a gene was upregulated in co-culture. The reverse is true in Swift, 2021.

**Genes upregulated in co-culture with *C. churrovis* as reported by Swift *et al.***			**EMBR-seq+ results (monoculture *vs* co-culture with *C. churrovis*)**				**EMBR-seq+ results (monoculture *vs* co-culture with *A. robustus*)**			
**Product name**	**Log2FC**	**Locus tag**	**Log2FC**	**P adj**	**Upreg. in monocult.**	**Upreg. in co-culture with C. churrovis**	**Log2FC**	**P adj**	**Upreg. in monocult.**	**Upreg. in co-culture with A. robustus**
ABC transporter ATP-binding protein	2.9	Ga0136279_2636	-1.53	1.58E-08		yes	-0.53	0.236		
Putative ABC transport system permease protein	2.2	Ga0136279_2635*	-0.59	7.34E-03			0.03	0.931		
Type II and III secretion system protein	2.2	Ga0136279_2405	-0.02	9.42E-01			0.47	0.136		
Outer membrane protein beta-barrel domain-containing protein	1.8	Ga0136279_1390	-1.08	2.53E-10		yes	-0.98	1.69E-04		yes
Urea ABC transporter	1.7	Ga0136279_1256	-0.62	3.81E-05		yes	-0.52	0.092		

substrate-binding protein										
Multispecies efflux RND transporter periplasmic adaptor subunit	1.6	Ga0136279_0657	-1.01	2.68E-08		yes	-0.50	0.124		
Zinc ABC transporter substrate-binding protein	1.6	Ga0136279_2085	-0.53	5.01E-01			-1.46	0.011		yes
Multispecies ammonium transporter	1.5	Ga0136279_1465	-0.99	1.14E-05		yes	-1.52	1.01E-07		yes
Transporter	1.5	Ga0136279_2620	-0.78	1.46E-04		yes	-0.30	0.299		
Multispecies ABC transporter substrate-binding protein	1.4	Ga0136279_2553	-0.55	3.64E-03		yes	-0.75	2.10E-03		yes
Iron complex outer membrane recepter proteina	1.4	Ga0136279_2080	1.95	1.27E-01			1.07	0.321		
General secretion pathway protein E	1.3	Ga0136279_1904	-0.71	6.92E-02		yes	-1.04	6.60E-03		yes
TonB family C-terminal domain-containing proteina	1.2	Ga0136279_1405*	0.59	5.87E-02			2.11	2.25E-12		
Multispecies efflux RND transporter periplasmic adaptor subunit	1.1	Ga0136279_1902	-0.67	8.75E-03		yes	-0.73	2.03E-03		yes

TRAP transporter large permease subunit	1	Ga0136279_0818	-0.34	1.32E-01			-0.19	0.619		
Calcium/sodium antiporter	1	Ga0136279_1391	-0.17	3.83E-01			0.12	0.675		
Multispecies TolC family protein	1	Ga0136279_1901	-0.49	5.44E-03			-0.44	0.057		

Supplementary Table S7. Upregulated CAZyme genes in monocultures of *F. succinogenes* strain UWB7 *vs*. co-cultures of *F. succinogenes* strain UWB7 with *A. robustus* or *F. succinogenes* strain UWB7 with *C. churrovis*. Monocultures and co-cultures were both grown on switchgrass.

	**Monoculture (*F. succinogenes* strain UWB7)**			**Co-culture (*F. succinogenes* strain UWB7 + *A. robustus*)**	
GH	GH5	Ga0136279_0422	GH	GH8	Ga0136279_1446
	GH5	Ga0136279_2901		GH10	Ga0136279_3005
	GH8	Ga0136279_1768		GH16	Ga0136279_0928
	GH9	Ga0136279_2267		GH5+CBM4	Ga0136279_1456
	GH9	Ga0136279_2903	GT	GT2	Ga0136279_1712
	GH10	Ga0136279_1559		GT2	Ga0136279_1713
	GH11	Ga0136279_0449		GT2	Ga0136279_1715
	GH23	Ga0136279_0889		GT2	Ga0136279_0012
	GH26	Ga0136279_2010		GT2	Ga0136279_0879
	GH74+GH74	Ga0136279_2365		GT2	Ga0136279_0852
	GH10+CBM6	Ga0136279_2195		GT4	Ga0136279_0554
	GH26+CBM35	Ga0136279_2970		GT4	Ga0136279_0639
PL	PL1	Ga0136279_2902		GT8	Ga0136279_0876
	PL1	Ga0136279_0674		GT32	Ga0136279_0638
CE	CE11	Ga0136279_1153		GT35	Ga0136279_0585
	CE15	Ga0136279_2918			
GT	GT2	Ga0136279_1342			

	**Monoculture (*F. succinogenes* strain UWB7)**			**Co-culture (*F. succinogenes* strain UWB7 + *C. churrovis*)**	
GH	GH5	Ga0136279_0422	GH	GH8	Ga0136279_1446
	GH5	Ga0136279_2901		GH16	Ga0136279_0928
	GH8	Ga0136279_1768		GH5+CBM4	Ga0136279_1456
	GH8	Ga0136279_1318		GH30+CBM6	Ga0136279_2169
	GH9	Ga0136279_2267		GH43+CBM6	Ga0136279_2166
	GH9	Ga0136279_2903	PL	PL1	Ga0136279_0688
	GH10	Ga0136279_1559	CE	CE6	Ga0136279_1612
	GH11	Ga0136279_0449		CE6+CBM6	Ga0136279_2170
	GH26	Ga0136279_2010		CE6+CBM6	Ga0136279_2171
GT	GT2	Ga0136279_1342	GT	GT2	Ga0136279_1712
CBM	CBM51	Ga0136279_2699		GT2	Ga0136279_1713
				GT8	Ga0136279_0876
				GT32	Ga0136279_0638
				GT35	Ga0136279_0585

REFERNCES

1. Swift CL, Louie KB, Bowen BP, Hooker CA, Solomon KV, Singan V, Daum C, Pennacchio CP, Barry K, Shutthanandan V, Evans JE, Grigoriev IV, Northen TR, O'Malley MA. 2021. Cocultivation of anaerobic fungi with rumen bacteria establishes an antagonistic relationship. mBio 12:e01442-21.

2. Wangsanuwat C, Heom KA, Liu E, O'Malley MA, Dey SS. 2020. Efficient and cost-effective bacterial mRNA sequencing from low input samples through ribosomal RNA depletion. BMC Genomics 21:717.

3. Kraus AJ, Brink BG, Siegel TN. 2019. Efficient and specific oligo-based depletion of rRNA. Sci Rep 9:12281.

4. Culviner PH, Guegler CK, Laub MT. 2020. A simple, cost-effective, and robust method for rRNA depletion in RNA-sequencing studies. mBio 11:e00010-20.

5. Huang Y, Sheth RU, Kaufman A, Wang HH. 2020. Scalable and cost-effective ribonuclease-based rRNA depletion for transcriptomics. Nucleic Acids Res 48:e20.

6. Engelhardt F, Tomasch J, Häussler S. 2020. Organism-specific depletion of highly abundant RNA species from bacterial total RNA. Access Microbiol 2:acmi000159.

7. Choe D, Szubin R, Poudel S, Sastry A, Song Y, Lee Y, Cho S, Palsson B, Cho B-K. 2021. RiboRid: A low cost, advanced, and ultra-efficient method to remove ribosomal RNA for bacterial transcriptomics. PLoS Genet 17:e1009821.

8. Prezza G, Heckel T, Dietrich S, Homberger C, Westermann AJ, Vogel J. 2020. Improved bacterial RNA-seq by Cas9-based depletion of ribosomal RNA reads. RNA 26:1069–1078.
